# Non-disclosing youth: a cross sectional study to understand why young people do not disclose suicidal thoughts to their mental health professional

**DOI:** 10.1186/s12888-021-03636-x

**Published:** 2022-01-04

**Authors:** Lauren McGillivray, Demee Rheinberger, Jessica Wang, Alexander Burnett, Michelle Torok

**Affiliations:** Black Dog Institute, University of New South Wales, Hospital Road, Randwick, NSW 2031 Australia

**Keywords:** Suicide prevention, youth suicide, disclosure, mental health professionals

## Abstract

**Background:**

Prevalence of suicidal ideation increases rapidly in adolescence, and many choose not to seek help and disclose their ideation. Young people who do disclose suicidal ideation, prefer to do so with peers and family compared to mental health professionals, who are best placed to provide evidence-based treatment. This study aimed to identify key factors associated with young people’s decision to, or not to disclose suicidal thoughts to their mental health practitioner.

**Methods:**

A community-based sample of young Australians (16 - 25 years), who had experienced suicidal ideation and engaged with a mental health professional, completed an online questionnaire (*N*=513) which assessed demographic characteristics, severity of depression, anxiety, psychological distress, and suicidal ideation, lifetime suicide attempts, exposure to suicide loss, personal suicide stigma, prioritisation of mental health issues, and therapeutic alliance. Logistic regression analyses were used to identify factors associated with disclosure.

**Results:**

Though the full sample had engaged in therapy, 39% had never disclosed suicidal ideation to their clinician. Those who had disclosed were more likely to report greater therapeutic alliance (OR=1.04, 95% CI=1.02–1.06), personal suicide stigma (OR=1.04, 95% CI=1.01–1.06), prioritisation of suicidal ideation (OR=.24, 95% CI=0.14-0.42), and lifetime history of suicide attempt (OR=.32, 95% CI=0.18-0.57). The most common reason for not disclosing was concern that it would not remain confidential.

**Conclusion:**

These findings provide new insights into why young people may not seek help by disclosing suicidal ideation, despite having access to a mental health professional, and establish evidence to inform practice decisions and the development of prevention strategies to support young people for suicide.

**Supplementary Information:**

The online version contains supplementary material available at 10.1186/s12888-021-03636-x.

## Background

Youth suicide prevention is a global priority as intentional self-harm is one of the leading causes of death amongst youth [1]. Suicidal ideation is an important target for youth suicide prevention efforts, with evidence that ideation increases the risk of a future suicide attempt in around one-third of young people [2]. Furthermore, suicidal ideation is one of the most common behaviours on the spectrum of suicidality, affecting around 15 to 29% of adolescents and young adults [3, 4]. Given increasing evidence demonstrating a pathway from ideation to suicide attempt [5, 6], the high prevalence of youth suicidal ideation is a matter of concern. While the best approach to treating suicidal ideation is through traditional therapeutic interventions (e.g., dialectical behavioural therapy and cognitive-behavioural therapy) delivered in clinical settings [7, 8], a number of barriers exist preventing indicated populations from accessing such services.

Of the young people who do access help and disclose their suicidal ideation, they primarily choose to do so with parents and peer groups rather than mental health professionals, such as psychologists, school counsellors) [9–11]. This preference of disclosing to social support networks occurs despite many young people having access to mental health professionals via school or other settings (see [12]). While disclosing to close family and friends is encouraged, members of these social networks often report they lack the confidence and skills to appropriately support the young person experiencing suicidal distress [13, 14]. As a result, this pathway can leave at risk young persons insufficiently supported [15, 16].

To date, there have been no studies which have examined the number of young persons who choose not to disclose their suicidal ideation, even when they have access to a mental health professional. Theoretically, there are a number of barriers that could feasibly prevent disclosure of suicidal thoughts and behaviours to clinicians, including perceived stigma [17, 18], difficulties communicating the need for help, concerns around a lack of privacy, mistrust of an unknown person, and beliefs that professional help will not be effective [19, 20]. Given that non-disclosure may prevent vulnerable young people from receiving targeted support for suicidal ideation – which has been shown elsewhere to require approaches that differ from depression or anxiety [21, 22] - it is important to understand the extent to which non-disclosure is an issue and why it occurs.

Accordingly, the key aims of this study are to:

1. Establish the prevalence of non-disclosure of suicidal ideation to a mental health professional among young people aged 16 to 25 years who are currently, or recently been, in treatment.
2. Identify the key reasons why young people decide not to disclose suicidal ideation to their mental health practitioner.

This novel study is the first the authors are aware of to explore the experience of, and reasoning behind, a young person’s decision to withhold disclosure of suicidal thoughts from mental health professionals. These insights could inform the development of policy and practice strategies to improve clinical care for young people with suicidal ideation.

## Methods

### Study design and recruitment

An online cross-sectional survey was designed and advertised to a community-based sample of young Australians. Ethics approval for this study was obtained from the University of New South Wales Human Research Ethics Committee (HC200465). STROBE guidelines [23] were used to report this study.

Potential participants completed a short screening survey to determine eligibility based on the following criteria: aged 16 to 25 years, living in Australia, fluent in English, had experienced suicidal thoughts in the past 12 months, and had engaged with a mental health practitioner for any reason in the past 12 months. Participants aged 16 and 17 years were considered mature minors that could participate without parental consent provided they had capacity to be involved [24], which was assessed through a Gillick Competency Task [25]. A copy of the questions and procedure to assess Gillick competency are presented in Appendix A. There were no study exclusion criteria.

The survey was published using the online survey platform Qualtrics on October 9th 2020, and data collection remained open until the recruitment objective was met (*N*=513) (12th October 2020). During this period, the survey was promoted on social media via targeted Facebook advertisements (target parameters: ages 16-25, Australia, interests in National Suicide Prevention, Lifeline, Suicide prevention, Beyondblue, Headspace, Lifeline, R U OK Day, SANE). When a potential participant clicked on the study advertisement, they were directed to an online study portal and asked to provide individual digital consent before completing a five-item screening survey to determine eligibility. Participants who did not meet the inclusion criteria (including the Gillick) were directed to a webpage thanking them for their time and which provided relevant support contacts, such as Kids Helpline and Lifeline phone numbers and webchat addresses. Participants who met the study inclusion criteria were directed to complete a 20-minute online self-report structured questionnaire, which also included free text options for questions relating to reasons why they chose whether or not to disclose suicidal thoughts to their mental health practitioner, and what would motivate them to disclose to a mental health professional in future. Participants who completed the survey were emailed a $10 e-gift voucher as compensation for their time. Relevant support contacts, such as Kids Helpline and Lifeline phone numbers and webchat addresses, were also provided on the consent form and at conclusion of the survey.

### Measures

Demographic characteristics were measured to describe the sample, including age, sex, sexual orientation, rural/remote or metropolitan location, relationship status, and mental health status. Sexual orientation was grouped into one of two categories for analyses: heterosexual or sexual minority (i.e., lesbian, gay, bisexual, queer, other, not sure, prefer not to say).

***Disclosure of suicidal ideation*** (primary outcome) was measured with the question, “Have you told your mental health professional that you have suicidal thoughts?” (‘yes’ or ‘no’). A follow-up open ended question was asked based on the response, that is “What factors made you choose to tell them you have suicidal thoughts?” or “What factors made you choose not to tell them you have suicidal thoughts?”. Open ended questions included multiple choice response options based on common reasons observed in the literature (e.g., concerns around a lack of privacy, mistrust of an unknown person, and beliefs that professional help will not be effective [19, 20];), as well as “other” - a free text response option. We also asked participants, “Can you tell us what sort of things would make you more likely to tell a mental health professional that you have suicidal thoughts?”. To compare the rate of disclosure to a mental health professional specifically with the rate of disclosure in general, we asked, “Have you ever told anyone about your suicide thoughts or behaviours?”.

***Suicidal ideation*** was measured using the Suicidal Ideation Attributes Scale (SIDAS, [26]). The SIDAS consists of 5 self-reported items rated on an 11-point scale (0 to 10). The scale provides a total score ranging from 0 to 50, with a higher score indicating greater suicidal ideation severity. Negatively worded items are reversed scored and scores of 21 or greater indicate a high risk for suicidal behaviour (attempt). The scale has demonstrated excellent internal consistency in the literature (α=.91, [26]) and good internal consistency in the present sample (α=.86).

***Depressive symptoms*** were measured by the Patient Health Questionnaire Depression Scale (PHQ-9, [27]). This scale consists of 9 items rated on a 4-point scale, ranging from 0 (not at all) to 3 (nearly every day). Higher scores indicate the presence of more depressive symptoms, and the maximum total score is 27. The scale has demonstrated good internal consistency in the literature (α>.80, [28]) and in the present sample (α=.86).

***Anxiety symptoms*** were measured by the Generalised Anxiety Disorder-7 Scale (GAD-7, [29]). The GAD-7 consists of 7 self-reported items rated on a 4-point scale (0 = not at all to 3 = nearly every day), with a total score ranging from 0 to 21 and higher scores indicating more severe anxiety symptoms. The scale has demonstrated good internal consistency in previous research (α>.80, [28]) and the present sample (α=.82).

***Psychological distress*** was measured using the Distress Questionnaire-5 (DQ5, [30]), which consists of 5 self-reported items rated on a 5-point scale (1 = never to 5 = always). The scale provides a total score ranging from 5 to 25, with a higher score indicating greater psychological distress. The scale has demonstrated good internal consistency in previous research (α=.86, [30]) and acceptable internal consistency in the present sample (α=.76).

***Suicide attempt*** (lifetime history) was assessed with the question “Have you ever attempted suicide?” (‘no, never’, ‘yes, once’, or ‘yes, more than once’). For participants who answered more than once, an additional question asked them to specify the number of attempts.

***Exposure to suicide loss*** was measured by asking, “Has anyone close to you died by suicide?” ‘yes’ or ‘no’. This question was a modified from a recent study by Maple and Sanford [31].

***Personal suicide stigma*** was measured using the Personal Suicide Stigma Questionnaire (PSSQ, [32]), which consists of 16 self-reported items rated on a 5-point scale (1 = never to 5 = very often). The scale provides a total score ranging from 16 to 80, with a higher score indicating suicide-related stigma experiences. The scale has demonstrated excellent internal consistency in previous research (α=.96, [33]) and the present sample (α=.92).

***Prioritisation of mental health issues*** was measured with the question, “In the following list of mental health problems, we’d like you to rank the top 3, according to how important these are to you when talking to your mental health professional.”. Respondents could rank a list of ICD-10 categories of mental health diagnoses, and suicidal thoughts (see Appendix B), from 1 (most important) to 3 (less important). Prioritisation of suicidal ideation specifically (ranked as top 3; ‘yes’ or ‘no’), was used in regression analyses.

***Therapeutic alliance*** was measured using the Revised Helping Alliance Questionnaire (HAq-II, [34]), which asks respondents to carefully consider their relationship with their most recent therapist and rate 19 items on 6-point scale according to how strongly they disagree (1) to agree (6) regarding the mutual collaboration and bond between client and therapist. The scale provides a total score ranging from 19 to 114, with a higher score indicating greater alliance with the therapist. Negatively worded items are reversed scored. The scale has demonstrated excellent internal consistency in past research (α ≥.90, [35]) and the present sample (α=.93).

### Statistical Analysis

To detect non-disclosure of suicidal ideation in the cohort (primary outcome), a total minimum sample size of *n*=471 was needed. This estimate accounts for a youth population-level incidence of 12-month suicide ideation of 26% [3] and a 34% rate of suicidal ideation in a youth population engaged with mental health services [36], with power set at 90%, alpha set at 0.01.

Descriptive information was presented as proportions (%) and means (with standard deviation; SD). T-tests and chi-square (χ2) tests were conducted to establish whether the participants who did and did not disclose suicidal ideation differed significantly on demographic and clinical characteristics. Significant variables (with a more liberal cut-off of *p* <.10, see [37]) were chosen to be entered into a subsequent binary logistic regression model to examine what factors are independently associated with disclosure of suicidal ideation to a mental health professional. In this model, disclosure (yes/no) as the dependent variable was used to estimate odds ratios (ORs) with 95% confidence intervals (CIs).

Quantitative data were analysed using SPSS version 25.0, alpha was set at *p* < .05 for interpreting significant effects. The following guidelines were used to interpret effect sizes: correlation coefficient (r) values of .10, .30, .50, and .70, correspond to small, medium, large, and very large effect sizes, respectively [38]; Phi (ϕ) values of .10, .20, .30, and .40, correspond to small, medium, large, and very large effect sizes, respectively [39]; ORs of 1.44, 2.48, and 4.27 correspond to small, medium, and large effect sizes, respectively [38]; raw means were calculated to estimate Cohen’s d, with values of .20, .50, and .80 corresponding to small, medium, and large effect sizes, respectively.

Responses to the three multiple-choice options "other” allowed respondents to provide free-text responses, which were analysed thematically using NVivo version 12. Using an inductive approach, responses were reviewed independently by two authors (LM and DR), with similar responses grouped together. Groupings were then reviewed by LM and DR together and discrepancies were discussed until agreement was reached, to form a single set of themes for each of the three free-text questions.

## Results

A missing values analysis found that less than 5% of values were missing for variables under investigation, and that they were missing completely at random (Little's MCAR test: *p* = .125). Therefore, it was safe to list-wise removes cases (SPSS default) and report on the ‘valid percent’ in descriptive statistics.

### Descriptive statistics

In total 513 participants completed the survey, with a median age of 17 years. The majority of participants reported being diagnosed with a mental disorder (*n*=412, 80.3%). Other demographic characteristics are displayed in Table 1.

**Table 1:** Sample Characteristics and Disclosure of Suicidal Ideation to a Mental Health Professional

	Total sample	No disclosure	Disclosure	Significance test and effect size
Sex				
Female	429 (83.8%)	161 (84.3%)	251 (83.9%)	χ^2^(1) .01, *p*=.919, ϕ=.005
Male	83 (16.2%)	30 (15.7%)	48 (16.1%)	
Sexuality				
Heterosexual	161 (31.4%)	78 (40.8%)	74 (24.7%)	χ^2^(1) 14.10, *p*<.001*, ϕ=.170
Sexual minority	352 (68.6%)	113 (59.2%)	225 (75.3%)	
Language at home				
English only	460 (89.7%)	173 (90.6%)	267 (89.3%)	χ^2^(1) .21, *p*=.648, ϕ=.021
Multilingual	53 (10.3%)	18 (9.4%)	32 (10.7%)	
Relationship status				
Single/not dating	367 (71.5%)	148 (77.5%)	202 (67.6%)	χ^2^(1) 5.63, *p*=.018, ϕ=.107
Partner/dating	146 (28.5)	43 (22.5%)	97 (32.4%)	
Location				
Metropolitan	401 (78.5%)	153 (80.1%)	233 (78.5%)	χ^2^(1) .19, *p*=.661, ϕ=.020
Rural/remote	110 (21.5%)	38 (19.9)	64 (21.5%)	
Prioritisation of suicidal ideation				
No	209 (40.7%)	112 (58.6%)	78 (26.1%)	χ^2^(1) 52.02, *p*<.001*, ϕ=.326
Yes	304 (59.3%)	79 (41.4%)	221 (73.9%)	
Lifetime suicide attempt				
No	273 (53.6%)	137 (71.7%)	123 (41.1%)	χ^2^(1) 43.79, *p*<.001*, ϕ=.299
Yes	236 (46.4%)	54 (28.3%)	176 (58.9%)	
Exposure to suicide loss				
No	416 (81.9%)	161 (84.7%)	237 (79.3%)	χ^2^(1) 2.30, *p*=.130, ϕ=.069
Yes	92 (18.1%)	29 (15.3%)	62 (20.7%)	
Disclosed to anyone				
No	91 (18.1%)	80 (41.9%)	5 (1.7%)	χ^2^(1) 131.45, *p*<.001*, ϕ=.518
Yes	413 (81.9%)	111 (58.1%)	294 (98.3%)	
Age (years M, SD)	17.6 (1.9)	17.36 (1.83)	17.71 (2.05)	t(488) = -1.91, *p*= 0.057, d= .191
Depression^a^ (M, SD)	27.45 (5.33)	26.99, 5.02	27.94, 5.43	t(488) = -1.94, *p*= 0.053, d= .189
Anxiety^b^ (M, SD)	20.31 (4.97)	20.48, 4.86	20.29, 5.04	t(488) = .41, *p*= 0.679, d= .039
Distress^c^ (M, SD)	19.04 (3.54)	18.98, 3.45	19.17, 3.52	t(488) = -.60, *p*= 0.547, d= .055
Suicidal ideation^d^ (M, SD)	12.51 (8.75)	10.41, 7.86	13.85, 8.97	t(482) = -4.30, *p*< 0.001*, d= .438
Personal suicide stigma^e^ (M, SD)	51.20 (13.89)	46.66, 14.46	53.12, 13.22	t(382) = -4.23, *p*< 0.001*, d= .447
Therapeutic alliance^f^ (M, SD)	79.76 (17.84)	74.20, 16.88	83.23, 17.58	t(462) = -5.46, *p*< 0.001*, d= .329

*p< .003 denotes significance after Bonferroni adjustment

^a^Measured with the Patient Health Questionnaire Depression Scale (PHQ-9, [28]).

^b^Measured with the Generalised Anxiety Disorder-7 Scale (GAD-7, [29]).

^c^Measured with the Distress Questionnaire-5 (DQ5, [30]).

^d^Measured with the Suicidal Ideation Attributes Scale (SIDAS, [26]).

^e^Measured with the Personal Suicide Stigma Questionnaire (PSSQ, [32]).

^f^Measured with the Revised Helping Alliance Questionnaire (HAq-II, [34]).

Most participants reported having disclosed suicidal thoughts to another person (*n*=413, 81.9%), however when asked if they had disclosed to a mental health professional more than one third said they had not (*n*=191, 39%). In ranking the importance of different mental health issues when talking to a mental health professional, 304 (59.3%) participants prioritised suicidal ideation as being important, but depressive (*n*=349, 68%) and anxiety-related disorders (*n*=328, 63.9%) were prioritised as the top two concerns. Some participants reported that they had never disclosed suicidal thoughts to anyone at all (*n*=80, 16.1%). Other clinical characteristics are displayed in Table 1.

### Characteristics of non-disclosure

Sample characteristics by disclosure status (disclosure, non-disclosure) are reported in Table 1 alongside significance testing and effect sizes. Disclosure was significantly associated with sexual minority status, prioritisation of suicidal ideation, a history of suicide attempt, having disclosed to another person in the past, greater suicidal ideation severity, greater personal suicide stigma, and greater therapeutic alliance with their most recent therapist.

Bivariate correlations were calculated between all variables under consideration (17 variables). After making Bonferroni corrections (*p* <.003), disclosure of suicidal ideation to a mental health professional (yes) had a large significant association with disclosure to anyone (*r*=.518, *p*=.000), a medium association with prioritisation of suicidal ideation (*r*=.326, *p*=.000), history of suicide attempt (*r*=.305, *p*=.000), and small associations with greater therapeutic alliance (*r*=.246, *p*=.000), greater personal stigma of suicide (*r*=.211, *p*=.000), greater suicidal ideation severity (*r*=.192, *p*=.000), and sexual minority status (*r*=.170, *p*=.000).

Results from the binary logistic regression are presented in Table 2. ‘Disclosure of suicidal ideation to anyone’ was not included in the model as an independent variable due to low cell frequency (i.e., *n*=5 had disclosed to anyone and not a mental health professional). The addition of eight independent variables to a model that contained only the intercept significantly improved the fit, χ2 (8) = 94.25, *p* < .001. The model explained between 22.8% (Cox and Snell R square) and 32.8% (Nagelkerke R square) of the variance in disclosure of suicidal ideation to a mental health professional. When compared to no disclosure, significant unique contributions to disclosure of suicidal ideation were made by prioritisation of suicidal ideation, personal suicide stigma, therapeutic alliance, and lifetime history of attempt. Participants that were significantly more likely to disclose suicidal ideation to a mental health professional prioritised suicidal ideation as one of the top three most important mental health issues to discuss (4.15 times greater odds than those who did not prioritise suicidal ideation in their top three ranking) and had a history of suicide attempt (3.15 times greater odds than those with no attempt history). Participants had greater odds of disclosing suicidal ideation to a mental health professional for every one-point increase on the therapeutic alliance scale (by 4.0%) and personal suicide stigma scale (by 3.7%).

**Table 2:** Binary Logistic Regression on Disclosure of Suicidal Ideation to a Mental Health Professional

Predictors	B	Wald χ^2^	OR	95% CI
Age	.15	3.67	1.16	0.99 to 1.36
Sexuality (heterosexual)	-.29	.95	.75	0.42 to 1.34
Suicidal ideation	.02	.87	1.02	0.98 to 1.06
Prioritisation of SI (yes)	-1.42	25.38***	.24	0.14 to 0.42
Depression	-.03	.94	.97	0.90 to 1.03
Personal suicide stigma	.04	8.50**	1.04	1.01 to 1.06
Therapeutic alliance	.04	23.78***	1.04	1.02 to 1.06
Lifetime attempt (yes)	-1.15	14.73***	.32	0.18 to 0.57

*p < .05; **p < .01; ***p < .001

### Reasons behind disclosure

Participant responses to questions relating to disclosure of suicidal ideation to a MHP are presented in Table 3.

**Table 3:** Reasons why participants chose to disclose, chose not to disclose, and what would motivate disclosure of suicidal ideation to a mental health professional

**Reasons for disclosing suicidal thoughts to their mental health professional**	
Response options	*n* (%)
I’m worried that suicidal thoughts will not go away, and I want help to manage them	153 (29.8%)
Suicidal thoughts are interfering with my life as much as/more than other mental health problems	152 (29.6%)
I believe that a mental health professional can help with my suicidal thoughts	125 (24.4%)
Someone I know told me I should tell my mental health professional	110 (21.4%)
Other reason/s (free text response)^a^	69 (13.5%)
I’ve talked about suicidal thoughts with a mental health professional in the past and found this helpful	52 (10.1%)
Suicidal thoughts have become a relatively new problem and I want to deal with them	38 (7.4%)
**Reasons for not disclosing suicidal thoughts to their mental health professional**	
I’m concerned that my mental health professional wouldn’t kept this information confidential	133 (25.9%)
Suicidal thoughts are not interfering with my life as much as other mental health problems are	81 (15.8%)
I think that suicidal thoughts will eventually go away, or are just a phase I’ll grow out of, so there’s no point talking about them	80 (15.6%)
I’ve not talked about suicidal thoughts with a mental health professional in the past, but think that they will judge me or not respond well	74 (14.4%)
Suicidal thoughts have become a normal part of my life	74 (14.4%)
I don’t think a mental health professional can do anything about my suicidal thoughts	65 (12.7%)
My mental health professional has never asked about suicidal thoughts so I’ve never brought it up	61 (11.9%)
I’ve talked about suicidal thoughts with a mental health professional in the past, was upset by their response, and don’t want to share again	25 (4.9%)
Other reason/s (free text response)^b^	18 (3.5%)
**Factors that would increase motivation to disclose suicidal thoughts to a mental health professional**	
If I knew this information would be kept confidential and not be shared with others	340 (66.3%)
If my mental health professional asked about suicidal thoughts	278 (54.2%)
If I thought that suicidal thoughts could be treated/ helped	274 (53.4%)
If I thought my mental health professional would respond without judgement	269 (52.4%)
If the suicidal thoughts became more frequent or severe	267 (52.0%)
If the suicidal thoughts started to interfere more with my work/ school/ relationships	241 (47.0%)
Other reason/s (free text response)^c^	20 (3.9%)

Each survey question allowed for endorsement of multiple response options.

^a^The most common themes were ‘Wanting to get help for their suicidal thoughts’ (*n*=25, 36.2%) and ‘Fear of current or future outcomes of acting on their suicidal thoughts’ (n21, 30.4%). Additional themes and exemplar quotes are provided in Appendix C.

^b^The most common themes were ‘Fear and consequences of disclosing’ (*n*=11, 61.1%) and ‘They were dismissive of their own thought’ (*n*=4, 22.2%). Additional themes and exemplar quotes are provided in Appendix B.

^c^The most common themes were, ‘Assurance of no hospitalisation’ (*n*=7, 35.0%) and ‘If they had a good relationship with their mental health professional’ (*n*=6, 30.0%). Additional themes and exemplar quotes are provided in Appendix C.

## Discussion

Although 100% of the current sample had reported experiencing suicidal ideation in the previous 12-months, we found that over one third (39%) chose not to disclose, despite having access to a mental health professional. This finding suggests that each year many young people at risk for suicidal behaviour are missing out on the opportunity to receive clinical care for suicidal ideation. Young people with access to a mental health professional are in the best possible position to receive effective evidence-based therapeutic intervention for their suicidal thoughts [40–42], so understanding why they choose not to disclose is critical to developing strategies to address this issue.

The findings in the present study showed that young people reported a number of barriers to disclosing suicidal ideation to a mental health professional that were different to those preventing young people from seeking help for mental health concerns, as noted in earlier studies (e.g., [18]). A hesitancy to disclose suicidal ideation was greater among those who did not have a suicide attempt history, potentially leading to a lower prioritisation of suicidal ideation as a key concern. Just over half of the participants (59%) ranked suicidal ideation as one of their top three most important mental health issues to discuss with a mental health professional, which was unexpected given that the entire sample had experienced suicidal ideation, compared to anxiety-related and depressive disorders, which were both ranked higher in priority. Consistent with this finding was that approximately half of the sample reported they would be motivated to disclose in future if their suicidal thoughts became more of a concern. Suicidal ideation is thought to exist on a continuum of ‘suicidality’, with less severe or ‘passive’ thoughts at one end and suicide attempts at the other [43]. However, the risk associated with suicide attempt and death is pronounced for suicidal ideation, independent of depression [44], particularly as many suicide attempts by young people are a result of impulse rather than planning linked to severity of ideation [45–47]. Therefore, while a certain a level of suicidal ideation has been considered ‘normative’ amongst adolescents [48], educating young people on the possible risk that suicidal ideation poses could encourage disclosure to mental health professionals.

Lower therapeutic alliance (less perceived closeness to and collaboration with the therapist, and lack of trusting the therapeutic process) was also associated with non-disclosure, as was the (false) belief that mental health professionals could not do anything to treat suicidal thoughts. Fewer positive expectations of treatment are typical amongst adolescents with depression and suicidal ideation, and this has been shown to predict future suicidal behaviour [49] and impede help-seeking [50]. The challenges of establishing a strong therapeutic alliance with adolescents and engaging them in therapy is well known [51]: youth are typically not self-referred and often enter into treatment unaware of their problems, and/or in conflict with their parents [52–54]. The success of establishing strong therapeutic relationships with young people is essential to improving their mental health outcomes [55] and may help to strengthen some of the motivations young people gave to disclose suicidal ideation in future (e.g., providing hope that suicidal thoughts can be treated/ helped, assurance that they would not receive negative judgement).

Gaining a young person’s trust has emerged as a significant aspect of the alliance development in adolescents, particularly by explaining limits of confidentiality [20, 56]. When participants were asked to indicate reasons for not disclosing suicidal ideation, the most reported reason was concern that their mental health practitioner would not keep this information confidential. The most common themes derived from free-text responses to reasons for not disclosing, and for motivating future disclosure, were ‘fear and consequences of disclosing’ (e.g., fear of being hospitalised) and ‘assurance of no hospitalisation’, respectively (see Appendix C). Taken together, these findings indicate fears around uncertainty of repercussions for some young people if they choose to disclose. In general, adolescent concerns around confidentiality have been well documented [19, 20]. Research in medical settings also indicates that young people's motivations to disclose sensitive information are impeded when confidentiality is not assured [57].

While psychologists practicing in Australia, America, and the UK are under no legal obligation to report this risk to others, unless there is an immediate and specified risk of harm to the young person that can be averted only by reporting this information [58–60], determining whether or not to breach confidentiality with a client in order to prevent harm can be a complicated and challenging process. Despite there being guidance about and support for confidentiality, ethical dilemmas and confusion amongst mental health professionals about when to breach confidentiality are widespread [61–63], particularly given the variation in mandatory reporting requirements within and between countries. Better training concerning confidentiality and ethical decision-making processes have been proposed as a necessary strategy to maintain the delicate balance of managing confidentiality in a way that respects adolescents’ developing autonomy, and protects the therapeutic relationship, while simultaneously protecting the young client from harm [64, 65]. Training may also prompt clearer discussions with young people about the basic rules of confidentiality, what information is protected, and the limits to confidentiality (e.g., mandatory reporting). Doing so may alleviate unrealistic fears held by the young person and mitigate the likelihood of them choosing to conceal symptoms or forgo care altogether (as has been demonstrated in medical settings [66–69];).

Concerningly, some young people reported not disclosing suicidal thoughts to their mental health practitioner because they were not directly asked. Previous studies have shown that health care providers do not routinely ask about suicide-related behaviours (which includes ideation, [70, 71]), with a discomfort in asking being identified as a common reason why [71–73]. Training in how to use screening tools and in how to help individuals identified at risk of suicide has been shown to increase clinician’s comfort and confidence in asking [71, 72]. Mental health professionals may be reassured to know that asking does not cause harm or increase suicide risk [74] and may benefit young people emotionally [75]. It may also help to address non-disclosure and associated suicide risk of young people seeking treatment if mental health professionals incorporate into their continual professional development plan, activities and practices to enhance their therapeutic relationships with young clients (e.g., see [76, 77]) and the quality and scope of their risk assessments. For example, conducting periodic assessments of suicide risk over the course of treatment, and not just during the initial session, and monitoring this risk is necessary because suicidal ideation can fluctuate dramatically [78, 79]. Moreover, training in conducting evidence-based interventions and ongoing suicide risk assessments may increase provider confidence in handling suicide risk while also increasing the young person’s exposure to – and potential comfort in answering these questions [80]. Unfortunately, there will be a proportion of young people who choose not to disclose to anyone at all (16.1% in the current sample); future qualitative work with this group is needed to understand why and then determine whether these insights could guide the development of policy or interventions that encourage them to understand when it is important to disclose.

Interestingly, reports of greater personalised suicide stigma were associated with disclosing suicidal ideation to a mental health professional. This finding is counter intuitive, as suicide stigma has been found to be a reported barrier to young people seeking help for mental health problems [17, 18]. As most of the items on the Personalised Suicide Stigma Scale focus on perceived rejection from friends and family following disclosure - rather than health professionals - this scale appears to be a measure of stigma resulting from negative interactions with informal supports, specifically. Therefore, an explanation for our finding might be that a greater degree of personalised suicide stigma, resulting from negative interactions with informal supports, leads to a desire to disclose suicidal ideation to formal support as an alternative when seeking help for suicide. Future research should examine whether different types of stigma (of which there are at least eight, see [81]) influence pathways for young people seeking help for and disclosing suicidal thoughts and behaviours.

### Limitations

There are several limitations that should be acknowledged. First, there were a disproportionate number of females (84%) and participants identifying as having a sexual minority status (69%) compared to what is represented in the general Australian population, at 50.7% [82] and 4% [83], respectively. However, considering our inclusion criteria to participate, this profile was not surprising as young females report experiencing higher rates of suicidal ideation [84] and help-seeking for help for mental health problems than males [85] (This trend is also seen amongst sexual minority youth compared to their heterosexual peers [86] Given the exploratory nature of this study, imbalances in the sample are less of threat to validity than confirmatory studies. Second, as most of our sample were aged 17 years, the findings may not fully reflect the experience of young people outside of this age range. Third, the study was based on cross-sectional data and causal relationships cannot be determined. Fourth, the pre-determined answers on our questionnaire may have led participants, and though we did include free text responses, qualitative interviews or a mixed-methods approach would have provided a richer understanding of the topics explored. Fifth, the sample size was not large enough to conduct sub-analyses (e.g., to examine whether confidentiality was more of a concern for young people under 18 years). Sixth, the accuracy of self-report measures is limited by honesty and introspective ability of respondents, and a multiple method approach to data collection would be ideal. Last, there is no way of knowing if the participants had experienced suicidal thoughts while they were actively engaged with a mental health professional, as both were asked about ‘in the past year’.

## Conclusions

Young people with access to a mental health professional are best placed to receive treatment for their suicidal thoughts, yet this study shows that more than one-third of young people choose not to disclose suicidal thoughts to mental health practitioners. These participants were more likely to report no history of suicide attempts, a low priority of their suicidal thoughts, lower therapeutic alliance, and lower personal suicide stigma. Several strategies for improving disclosure to mental health professionals were discussed, including ways to psycho-educate young people on why disclosing ideation is important, and allaying fears around uncertainty of repercussions if they choose to disclose. For clinicians, training in how to conduct ongoing risk assessments over the course of treatment – including how to directly ask about ideation and respond to suicide risk - will be important to implement in tandem with any strategies directly focused on young people, to create a systems response to creating safety to disclose.

## Supplementary Information

**Additional file 1.**

**Additional file 2.**

**Additional file 3.**

## Data Availability

The datasets generated and/or analysed during the current study are not publicly available due to the sensitive nature of the data collected from minors but are available from the corresponding author on reasonable request.
