# Supplementary material for: Non-disclosing youth: a cross sectional study to understand why young people do not disclose suicidal thoughts to their mental health professional
Source: BMC Psychiatry. 2022 Jan 4;22:3. doi: 10.1186/s12888-021-03636-x (PMC8728900; doi:10.1186/s12888-021-03636-x)
Supplement: Supplementary file 1 — Additional file 1. [file 12888_2021_3636_MOESM1_ESM.docx]

**Appendix A:** Gillick Competency Task

**Q1: What is this study about?**

o Understanding why young people disclose or don’t disclose suicidal thoughts to their mental health professional/s (1)

o Evaluating if a mental wellbeing program is effective or not (0)

o I have no idea (0)

**Q2: How old do you have to be to participate in this study?**

o Any age (0)

o 16-25 (1)

o I don’t know (0)

**Q3: What does the study involve?**

o Completing a screener survey and a questionnaire (1)

o Testing a mental-health mobile app (0)

o I don’t know (0)

**Q4: Do you feel pressured to participate in this study?**

o Yes (0)

o No (1)

o I’m not sure (0)

**Q5: This study is anonymous, which means that if you participate you will not be able to withdraw or access the information you provide us. Are you okay with this?**

o Yes (1)

o No (0)

Scoring: sum items 1-5.

Eligibility: scores = 5 are eligible and the following text appeared: "Great! Thanks for providing your consent. We will now ask you some questions about yourself to see if you’re eligible to go further."

Scores <5 are ineligible. The young person will be directed back and prompted to re-read the information sheet carefully, and try the test once more.

If ineligible after a second attempt the following text appeared: “Unfortunately, your responses indicate that you’re unable to participate in this study. Your interest and willingness to participate in this study is hugely appreciated. And just because this might not be the right study for you, it does not mean you are on your own.

If you require immediate support for your mental health, here are some suggestions:

• Lifeline: 13 11 14 (24-hrs) and webchat (7pm-midnight Sydney time): https://www.lifeline.org.au/get-help/online-services/crisis-chat

• Kids Helpline: 1800 55 1800 (24-hrs) and webchat (24-hrs): https://kidshelpline.com.au/get-help/webchat-counselling

• Suicide Callback Service: 1300 659 467 (24-hrs)

• If you are in immediate life-threatening danger, call 000.”
