# Supplementary material for: Non-disclosing youth: a cross sectional study to understand why young people do not disclose suicidal thoughts to their mental health professional
Source: BMC Psychiatry. 2022 Jan 4;22:3. doi: 10.1186/s12888-021-03636-x (PMC8728900; doi:10.1186/s12888-021-03636-x)
Supplement: Supplementary file 2 — Additional file 2. [file 12888_2021_3636_MOESM2_ESM.docx]

**Appendix B:** ICD-10 categories of mental health issues participants were asked to prioritise

Participants were asked, “In the following list of mental health problems, we’d like you to rank the top 3, according to how important these are to you when talking to your mental health professional.”. The following ICD-10 categories of mental health issues – and suicidal ideation – were presented:

- Suicidal thoughts
- Major Depressive Disorder or Depressive Disorder
- Bipolar Disorder
- Anxiety Related Disorder (e.g. Social Anxiety Disorder, Generalised Anxiety Disorder, Panic Disorder)
- Obsessive Compulsive Related Disorder
- Substance Related or Addictive Disorder (e.g. gambling, alcohol and other substance dependence)
- Eating Disorder
- Sleeping Disorder (e.g. Insomnia)
- Personality Disorder (e.g. Borderline Personality Disorder, Antisocial Personality Disorder)
- Post-Traumatic Stress Disorder
- Schizophrenia or Psychotic Disorder
- Neurodevelopmental Disorder (e.g. Autism Spectrum Disorder, Asperger Syndrome)
