# Supplementary material for: Non-disclosing youth: a cross sectional study to understand why young people do not disclose suicidal thoughts to their mental health professional
Source: BMC Psychiatry. 2022 Jan 4;22:3. doi: 10.1186/s12888-021-03636-x (PMC8728900; doi:10.1186/s12888-021-03636-x)
Supplement: Supplementary file 3 — Additional file 3. [file 12888_2021_3636_MOESM3_ESM.docx]

**Appendix C:** Themes coded from other/free-text responses to questions about disclosure of suicidal ideation to mental health professionals, alongside an exemplar quote.

| **“What factors made you choose to tell them (your mental health professional) you have suicidal thoughts?”** | | |
| --- | --- | --- |
| Themes | *n* | Exemplar quote |
| Wanting to get help for their suicidal thoughts | 25 | “I realised I need further help and was at rock bottom” |
| Fear of current or future outcomes of acting on their suicidal thoughts | 21 | “Fear of causing major damage to myself and surviving” |
| Participants felt forced do disclose by other people | 14 | “I was forced to by my parents at the time” |
| Participants were asked directly if they were experiencing suicidal thoughts by their mental health professional | 11 | “The mental health professional asked if I had ever experienced suicidal thoughts” |
| Participants had a good relationship with their mental health professional | 8 | “I told my mental health professional about my suicidal thoughts when I had a better relationship with her and trusted her with that information.” |
| Participants wanted to be honest and open with their mental health professional | 7 | “I wanted to reply honestly (although I was reluctant)” |
| Participants knew the session would be confidential | 4 | “I also did a lot of reading online before going into my session to confirm that she would not break confidentiality (unless I was in significant danger - which I was not), which made me feel more comfortable about telling her.” |
| Didn’t want to distress family or friends | 3 | “I was close to making another suicide attempt and didn’t want to hurt my family again” |
| Felt reassured by other’s experiences of disclosing suicidal thoughts | 1 | “I also read about other people's positive experiences with telling their psychologists which helped reassure me that they would not make a big deal out of it.” |
| **“What factors made you choose not to tell them you have suicidal thoughts?”** | | |
| Themes | *n* | Exemplar quote |
| Fear and consequences of disclosing | 11 | “Scared of hospital” |
| They were dismissive of their own thoughts | 4 | “I thought I (was) just being dramatic” |
| They felt judged when disclosing other mental health concerns previously | 1 | “Prior to her a lot of mental health professionals would tell me I was bad for being suicidal or ignore me when I tried to talk to them about my mental health” |
| Disclosing would restrict future career options | 1 | “My plan after university was to join the defence force and if they know I have had some thoughts of suicide, they would never accept me” |
| A lack of trust | 1 | “I didn’t tell due to trust issues” |
| The mental health professional was not available | 1 | “This is very recent (last month or so), and my mental health provider has been unavailable since before suicidal thoughts occurred” |
| They didn’t feel that suicidal thoughts were relevant to the problem they were engaged with the mental health professional for | 1 | “The professional was for being transgender not specifically depression” |
| They believed suicidal thoughts were normal | 1 | “When I was really depressed and I thought about killing myself daily I didn't even think I was depressed, I assumed it was normal to feel like that.” |
| They felt the suicidal thoughts were under control | 1 | “I had those thoughts under control” |
| **“Can you tell us what sort of things would make you more likely to tell a mental health professional that you have suicidal thoughts?”** | | |
| Themes | *n* | Exemplar quote |
| Assurance of no hospitalisation | 7 | “If I had their guarantee that they wouldn't send me to hospital” |
| If they had a good relationship with their mental health professional | 6 | “If the clinician is kind and has my trust already” |
| If they knew it would be helpful to disclose | 5 | “If I knew how it would benefit my life/state of mind and wellbeing.” |
| If they were less fearful of disclosing | 2 | “If I had the courage to bring it up.” |
| They would not disclose for any reason | 2 | “I really don’t think I’ll ever say it.” |
| If the mental health professional was available | 1 | “If my mental health professional was available.” |
| Note: Participant responses to each question can be coded across multiple themes related to that question. | | |
